# Impact of the Quadriceps Angle on Health and Injury Risk in Female Athletes

**DOI:** 10.3390/ijerph21121547

**Published:** 2024-11-22

**Authors:** Hannah Gant, Nabin Ghimire, Kisuk Min, Ibrahim Musa, Maryam Ashraf, Ahmed Lawan

**Affiliations:** 1 Department of Biological Sciences, University of Alabama in Huntsville, SST 369H, 301 Sparkman Drive, Huntsville, AL 35899, USA; hrg0009@uah.edu (H.G.); ng0063@uah.edu (N.G.); ma0164@uah.edu (M.A.); 2 Department of Kinesiology, University of Texas at El Paso, El Paso, TX 79968, USA; kmin@utep.edu; 3 School of Sport, Exercise and Rehabilitation Sciences, University of Birmingham, Birmingham B15 2TT, UK; ibrophs83@gmail.com

**Keywords:** quadriceps angle, sports injury risk, female athletes

## Abstract

The quadriceps angle, knowns as the Q-angle, is an anatomical feature of the human body that is still largely unknown and unstudied despite its initial discovery in the 1950s. The strength disparities between male and female athletes are largely determined by the Q-angle. In spite of a growing number of women participating in sports such as track, tennis, soccer, gymnastics, basketball, volleyball, swimming, and softball, studies investigating injuries in this group are scanty. Even though the Q-angle has been the subject of many studies carried out all over the world, a review of the literature regarding its effects on health and injury risk in female athletes has not yet been completed. The aim of this review is to examine the crucial role of the Q-angle in the biomechanics of the knee joint and its effect on performance and injury risk, particularly in female athletes. Furthermore, we highlight the greater likelihood of knee-related injuries seen in female athletes being caused by the Q-angle. Athletes, coaches, healthcare professionals, and athletic trainers can better comprehend and prepare for the benefits and drawbacks resulting from the Q-angle by familiarizing themselves with the research presented in this review.

## 1. Introduction

The human body consists of many complex systems. Each component of the system cooperates with others to enable the body to function. The muscular system and the skeleton system rely heavily on each other. There would be no attachment sites for the muscles if there were no skeletal system. The body can move with the aid of nerve impulses because of the tendon connections that link muscles to bones [1,2]. The human body as a whole is a meticulously constructed machine, with each system influencing the others. A key illustration of this coordination is the quadriceps (Q) angle. The aim of this review is to examine the crucial role of the Q-angle in the biomechanics of the knee joint and its effect on performance and injury risk, particularly in female athletes.

**Figure 1:**
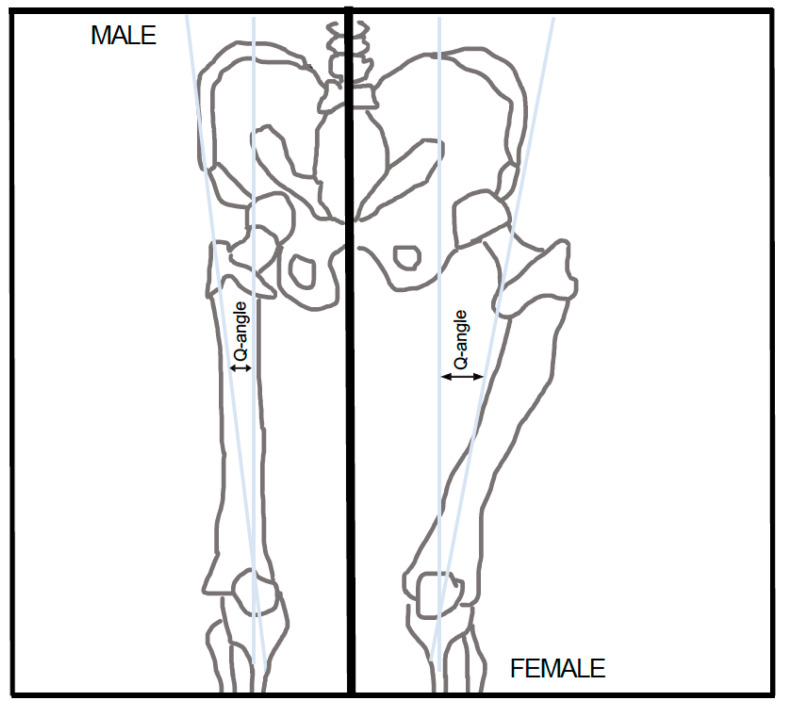
Difference between the Q-angle in the male and female skeletal structure: 14º for males and 17° for females.

The quadriceps angle, also known as the Q-angle, as first defined by Brattstroem et al. [3] in 1964, is the angle formed by the line created by the anterior superior iliac spine (ASIS) and the midpoint of the patella intersecting with the line formed by the patella midpoint and the tibial tubercle [3]. Another way to define this angle is to determine the angle between the anatomical and mechanical axes of the femur [4]. This angle varies from person to person and from sex to sex, with an average angle of 14° for males and 17° for females [5]. This difference in male and female Q-angles is illustrated in Figure 1.

Lower Q-angles are associated with taller subjects [6]. This is because taller individuals have longer femurs, which help to decrease the Q-angle. Because males are generally taller than females, males tend to have lower Q-angles. Although females have a wider pelvis than males, the ASIS in males and females is the same, with neither sex having a more lateralized ASIS than the other. Despite these anatomical characteristics, the theory that women’s wider pelvises contribute to their higher Q-angle is still widely accepted.

## 2. The Anatomy of the Q-Angle

The Q-angle has a direct impact on the knee and the ankle and is the most frequently used to identify lower limb malalignment [2]. While the Q-angle refers to the angle formed between the ASIS of the ilium and the midline of the patella, it influences the angle of attachment for the quadriceps muscle and the patellar tendon [1]. The alignment of the hips in relation to the lower extremity impacts how weight is distributed on the joints and can place unnecessary strain on certain ligaments and parts of the bones [2]. This alignment impacts the alignment of inferior areas, such as the foot and ankle [2]. The Q-angle influences muscles and tendons that play crucial roles in the biomechanics of the leg [6]. For example, an abnormally large Q-angle alters the movements of the patellofemoral joint and increases the lateral traction placed on the patella by the quadriceps group. Another example of the Q-angle’s impact on the lower limb is the significant relationship between an increased Q-angle and overpronation of the ankle [6]. Despite being a measurement of the hip to the knee, the Q-angle has an impact on the entire lower extremity, ranging from strength to health.

To better understand how the Q-angle affects the knee, we must first understand the anatomy and simple concepts of the biomechanics of the knee. The knee is composed of bones, including the femur, the patella, and the tibia [7]. Additionally, it contains both articular and meniscus cartilage. However, the anatomy of the knee that the Q-angle impacts the most is the ligaments of the knee. The knee contains two types of ligaments: the cruciate ligaments and the collateral ligaments [3,7]. The collateral ligaments are responsible for stability in the medial and lateral directions. The knee has two collateral ligaments: the medial collateral ligament (MCL,) which provides valgus stability, and the lateral collateral ligament (LCL), which supports the lateral side of the knee joint [8]. The cruciate ligaments provide stability in the frontal and posterior directions. The two cruciate ligaments of the knee are the anterior cruciate ligament (ACL) and the posterior cruciate ligament (PCL) [9]. All of these components—bones, cartilage, and ligaments—work together to improve the overall stability of the joint, hold the knee together, and work with the quadriceps and hamstring muscles to create skeletal movement [9].

The Q-angle affects the anatomy of the knee by altering the biomechanics and altering the distribution of weight on the knee joint [5,10]. Females typically have a larger Q-angle than males [10]. The problem with this is that a larger Q-angle causes the knees to bend inward and displaces more weight and force on the lateral side of the knee [5]. By displacing more force on the lateral side of the knee, the Q-angle places more stress on the MCL and the ACL. Over time, this stress can weaken these ligaments and reduce the strength and stability of the knee. This additional stress could potentially lead to partial or total ligament tears [11]. The Q-angle also affects the tracking of the patella [3]. Because the Q-angle impacts the angle at which the quadriceps muscles connect to the patella, the Q-angle impacts the direction in which the patella is being pulled [12]. The higher average Q-angle that females have means that the patella is pulled laterally. This lateral pull increases as flexion increases and can lead to the dislocation of the patella. The increased lateral stress placed on the knee joint and its component parts by the Q-angle leads to a higher risk of injury and an overall weaker knee [13].

In addition to altering the overall strength and stability of the knee, the Q-angle can also impact the ankle joint. The ankle joint is the point where the tibia, fibula, and talus bones meet [14]. The lateral border of the joint is formed by the articular facet of the lateral malleolus, while the articular facet of the medial malleolus forms the medial border [14]. The superior portion of the ankle joint is formed by the inferior articular surface of the tibia and the superior margin of the talus. The entire joint is surrounded by the articular capsule. In addition to the bones and the capsule, the ankle joint has multiple ligaments that aid in stabilizing the ankle. The deltoid ligament stabilizes the medial side of the ankle and is composed of four ligaments that connect the tibia to the navicular, calcaneus, and talus: the anterior and posterior tibiotalar ligaments, the tibionavicular ligament, and the tibiocalcaneal ligament [7,15]. In addition to the deltoid ligament, the anterior and posterior talofibular ligaments, as well as the calcaneofibular ligament, support the ankle joint laterally. The anterior and posterior talofibular ligaments connect the talus and the fibula to each other, while the calcaneofibular ligament connects the fibula and calcaneus [7,13]. These ligaments work together to strengthen the joint.

## 3. Q-Angle Measurement

There are two common methods of measuring the Q-angle; the static Q-angle and the dynamic Q-angle. When measuring the static Q-angle, we can choose to place the knee joint in one of two ways: either completely extended or with more than 20° of flexion when lying supine or upright [16,17]. Traditional or conventional measurement is performed while supine with the quadriceps relaxed and the knees extended [16,17]. Normally, in tasks involving closed kinetic chains (CKCs), the dynamic Q-angle is measured by recording the angle throughout the whole trajectory of motion [18]. The methods used in 3D kinematic analysis, such as photogrammetry or capture systems that use reflective markers in conjunction with camera motion, are incredibly accurate. On the other hand, dynamic knee valgus (DKV) is recorded on the frontal plane in 2D systems [9,18].

Reliability differs depending on how the Q-angle is calculated. In both adults and children, the conventional static Q-angle, which is measured with a goniometer on a plain radiograph, demonstrates low-to-moderate reliability [19,20]. However, using 2D (camera or MRI) and 3D motion analysis systems with cameras taking measurements yields excellent intra- and inter-rater reliability (ICC: 0.989 and 0.94–0.989, respectively) (system-based measurements) [19,20]. Similarly to the static Q-angle measurement using a goniometer (conventional measurement), the dynamic Q-angle measurement shows higher reliability (intra-rater ICC: 0.74–0.998; inter-rater ICC: 0.837–0.913) [18,21]. However, DKV values now indicate the true knee valgus [22]. Measuring Q-angles (static and dynamic) presents significant challenges [4,22]. As a result, opinions differ regarding the most accurate method for determining both static and dynamic Q-angles [23].

## 4. Impact of the Q-Angle on Athletic Performance

The skeletal system and the muscular system work together to generate skeletal movement. Because of this, bone structure can have effects on muscular strength and power output. This is why the Q-angle creates a biological advantage in sports. Due to its influence on the direction of force application, the Q-angle affects athletic performance and explains variations in quadriceps muscle gain [8]. Both a lateral force and a downward force (extensor force) are accounted for when the same amount of force is applied at the Q-angle. More force is applied laterally and less force is applied downward as the angle increases [24]. Therefore, a greater Q-angle leads to less force for the extension of the leg. This also means that less strength is produced by the quadriceps when compared to individuals with a smaller Q-angle [25].

The Q-angle can change the alignment of the tibia with respect to the femur [8]. An increase or a decrease in the Q-angle influences tibial rotation; external rotation is associated with an increased Q-angle [11]. This relationship works both ways though: the Q-angle can increase tibial rotation, while tibial rotation can impact the Q-angle. In most cases, the Q-angle leads to overpronation of the ankle. Overpronation has the most damaging effects on the subtalar joint, the location of articulation between the facets of the talus and the calcaneus [2,18]. During overpronation, the ligament that attaches the lateral malleolus and the talus remains taught, while the ligament that attaches the medial malleolus and the talus remains loose [23]. This places strain on the laterally attached ligament. In addition to placing strain on this lateral ligament, several pathologies are also associated with overpronation of the ankle. Some pathologies associated with ankle overpronation include Achilles pathology, tibialis posterior dysfunction, and patellofemoral pain, as well as an increase in the likelihood of knee injuries [23].

The Q-angle impacts the entire lower extremity in multiple ways. The Q-angle can lead to excess strain placed on specific ligaments of both the knee and the ankle. It can change the rotation of the foot, which is associated with several different lower limb pathologies [26]. The Q-angle can increase an individual’s likelihood of patellar subluxation or dislocation [11]. Despite being a measurement of the angle between the ASIS of the hip and the midline of the patella, the Q-angle impacts the entire anatomy of the knee and the ankle and can lead to knee injuries in athletes.

Top-tier athletes are built for competition. They have a genetic predisposition for either speed or strength. They were gifted with the perfect athletic build, height, or muscular density for their sport and have dedicated their lives to building upon what their DNA has given them and honing their skills [27]. Just like Michael Phelps’s wingspan and torso give him an advantage in swimming by increasing his power output, similarly, an athlete’s Q-angle can either help or hinder athletic performance in sports. This is due to the fact that the Q-angle affects how an athlete’s power output is distributed.

As previously mentioned, males and females have different average Q-angles of 14°and 17°, respectively. This means that male athletes can exert more extensor force than female athletes. Additionally, a greater Q-angle leads to overall decreased knee strength, power output, and torque angles [2]. As a result, the Q-angle raises the risk of injury and prevents female athletes from producing the same amount of power as male athletes. Trigonometry and physics can be used to demonstrate this difference [28]. Male athletes exert more force in the extension of the leg because their Q-angle is smaller, at 14° [28]. This improves the translation of the quadriceps muscles’ work into movement. Accordingly, female athletes exhibit a higher Q-angle of 17°, indicating that a greater proportion of their quadriceps work is displaced laterally [28]. This does not pose much of a difference at low levels or in single instances, but this difference can accumulate to impose a genetic difference between male and female athletes. As seen in Table 1, the female athlete must exert a larger amount of force in order to obtain the same amount of extensor force as their male counterparts. This difference continues to grow as weight increases. In power sports, such as weightlifting and powerlifting, this difference could account for a portion of the performance difference seen between male and female athletes [26]. In sports such as running, this difference in power output can accumulate with each stride and could account for differences observed in male and female competitors’ times [26].

All activities’ biomechanics are significantly influenced by the Q-angle, but some sports are more affected by it than others. The Q-angle influences one sex more than the other and is more significant for certain sports than others. Every sport uses the Q-angle, but postpuberty female athletes’ athletic performance is where it really stands out [14]. Until puberty, male and female adolescents have similar average Q-angles [26]. Children between ages 7 and 12 have no significant differences in Q-angle, with young males having an average Q-angle of 15.7° and young females having an average Q-angle of 15.6° [26]. These values mean that young males and young females have similar levels of power output and force distribution, which allows for similar levels of performance. This can be seen in the similar sprinting speeds of young athletes [11]. These values begin to differ once females reach puberty. When a female goes through puberty, she goes through a series of events and changes. One of these changes is the widening of the hips. As the hips widen, the Q-angle increases. Because a higher Q-angle correlates with a smaller extensor force, puberty negatively impacts female athletes [14]. This means that most female athletes notice a decline in their athletic capabilities very early on in life. This phenomenon is very well known in the world of running [26]. In addition to the Q-angle slowing down young female runners, the widening of the hips also places more stress laterally on the knee and increases the risk of an ACL injury.

## 5. Effect of the Q-Angle on Injury Risks

The Q-angle plays a crucial role in the biomechanics of the knee joint, and its effect on injury risks, particularly in female athletes, is a topic of interest in sports medicine and biomechanics. Large Q-angles are associated with lower extremity injuries in military training, overuse injuries in other sports populations, and patellofemoral pain and other running-related injuries in adolescents and adult competitive and recreational runners [29].

## 6. Patellofemoral Pain Syndrome (PFPS)

Patellofemoral pain syndrome (PFPS), commonly known as runner’s knee or jumper’s knee, is characterized by pain around or behind the patella (kneecap), especially during activities that involve bending the knee, such as running, jumping, or squatting [30,31]. While the exact cause of PFPS is multifactorial and still not fully understood, the association between a wider Q-angle and increased risk of PFPS has been a topic of research interest [32,33,34]. The increased Q-angle causes lateralization of the patella, which can cause maltracking and excessive stress on the patellofemoral joints during sports. The maltracking of the patella is caused by the delayed muscle activity of the vastus medialis oblique (VMO) muscle relative to the vastus lateralis (VL) [35]. However, it is important to note that the relationship between the Q-angle and PFPS is not entirely straightforward, and findings from studies investigating this association have been scanty. The study by Hvid and Andersen [36], which observed a significant difference in mean Q-angle values between males and females with patellofemoral diseases, provides further insight into the potential role of the Q-angle in PFPS among female athletes [37]. The exact cause of PFPS is still unknown, but research has shown a significant difference in mean Q-angle values between males and females with patellofemoral diseases, providing further insight into the potential role of the Q-angle in PFPS.

## 7. Anterior Cruciate Ligament (ACL) Injuries

An ACL tear is the most severe knee ligament injury in sports [38]. ACL tears are more prevalent among female athletes compared to male athletes [39]. Some research indicates that a larger Q-angle may be associated with an increased risk of ACL injuries, especially in female athletes, even though the relationship between the Q-angle and ACL injuries is not as clear-cut as it is with PFPS or patellar instability [38,40]. The increase in Q-angle among females is related to several anatomical factors, including increased pelvic width, shorter femur length, or more laterally placed tibial tuberosity [41]. Understanding these anatomical differences and their relationship to the Q-angle can help sports medicine clinicians and researchers better assess and manage ACL conditions.

## 8. Q-Angle and Patellar Dislocation

The increase in Q-angle is linked with patellar dislocation. A study of in vitro knee simulation to determine the relationship between the Q-angle and patella kinematics showed that the increase in Q-angle led to significant lateral shifting (20° to 60°), medial tilting (20° to 80°), and medial rotation (20° to 50°) of the knee flexion [32]. This study suggests that the increase in Q-angle is a risk factor for lateral patellar dislocation. An increase in Q-angle is also one of the factors causing PFPS [42].

## 9. Q-Angle and Ankle Injuries

The Q-angle may be a risk factor for ankle injuries. A study of young men who had completed 12 weeks of army infantry training found that those with a valgus knee and a Q value greater than 15 were at increased risk of suffering injuries in the lower leg [43]. Another study of 45 subjects showed a positive relationship between Q-angle and ankle sprains in recreational basketball players [44]. Rauh et al. (2007) studied the role of the Q-angle in lower extremity injury in 393 high school cross-country runners. The study found that runners with a Q-angle greater than 20° were 1.7 times at greater risk of injury than runners with a Q-angle less than 15° [45]. In another study of 300 patients with ankle sprains, it was found that Q-angle significantly correlated with a history of ankle sprains. The Q-angle was wider by 2° in patients with a history of ankle sprains [46]. More studies are needed to establish the effect of age, BMI, and Q-angle in the context of ankle sprains.

## 10. Combatting the Risks and Setbacks Related to the Q-Angle

It is a fact that female athletes who participate in the same sports as their male counterparts are four to six times more likely to sustain injuries. Anatomical variations account for the majority of injuries, with the Q-angle being the most important (Ref. Hutchinson, M.R., 1995). Consequently, there is a significant chance of injury reduction for female athletes if these anatomical structures are strengthened.

## 11. Athletes Can Strengthen and Stretch the Surrounding Muscles to Stabilize the Knee Joint

Athletes can strengthen and stretch the surrounding muscles to stabilize the knee joint. Exercises targeting the quadriceps, hamstrings, hip flexors, and calf muscles can help reduce strain and pressure on the knee joint, alleviating pain and stabilizing it [2]. A comprehensive program targeting the muscle group around the knee can effectively stabilize the knee joint [47]. Hip and knee strengthening exercises are more effective than knee strengthening exercises alone in decreasing pain and improving activity in the case of patellofemoral pain [48,49]. Eight weeks of an isokinetic muscle strength program among study subjects with osteoarthritis of the knee joint showed a significant decrease in pain and stiffness and increased mobility compared to the control group [50]. Also, it has been reported that isokinetic training, which involves maximal contraction at a constant angular velocity, is effective in knee joint function and minimizes DKV, which can prevent lower limb injuries in athletes [51]. Similarly, other studies have reported that isokinetic muscle strengthening significantly reduces pain and provides dynamic muscle strengthening for knee osteoarthritis [52].

## 12. Strengthening the Vastus Medialis Oblique (VMO) Muscle 

The VMO muscle is the most medial portion of the quadriceps muscles [53]. The fibers of the VMO run obliquely and are inserted directly into the upper border of the patella bone. Consequently, the primary function of the VMO muscle is medial stabilization of the patella during knee extension [54]. Weakness or imbalance in the VMO muscle results in patellar maltracking, where the patella deviates laterally during movements including squatting, jumping, or running [55,56]. This patella tracking disorder can increase the risk of patellofemoral pain syndrome and other knee injuries [56]. Therefore, strengthening the VMO muscle is effective in stabilizing the knee joint and preventing injury in female athletes with an abnormal Q-angle. A study by Elias et al. (2009) found that an increase in the force applied by the VMO and other quadriceps muscles decreased the lateral pressure, suggesting that improving VMO function reduces the pressure applied to the lateral cartilage in the patellofemoral joint [57]. A study of 21 male participants found that the six weeks of a quadriceps femoris strengthening exercise program significantly increased the VMO fiber angle and insertion length [58]. The exercises that strengthen the VMO include terminal knee extensions, short arc quad exercises, quad sets, and wall squats with a ball [59,60].

A semi-squat exercise with hip adduction can increase the activity of the VMO and VLL muscles, which helps to balance the activation of the medial and lateral quadriceps in PFPS patients [61]. Similarly, sling-based closed kinetic knee extension exercises significantly activate the VMO compared to hip adduction exercises and can benefit PFPS [62]. In contrast, some studies suggest targeting the quadriceps muscle is more beneficial to improving the knee joint. A study on the effect of two exercises, CKC and open kinetic chain (OKC) exercises, on the onset of electromyographic (EMG) activity of the quadriceps muscles found that CKC exercise promotes simultaneous activation of the quadriceps muscles compared to OKC [63]. Closed kinetic chain exercise can be used in designing rehabilitation and training programs to target quadriceps muscles and control the patellofemoral joint [63].

## 13. Strengthening the Quadriceps Muscles

The Q-angle is the angle between the quadriceps muscle tendon and the patellar tendon [64]. The Q-angle is used to evaluate the biomechanical alignment of the knee joint and influence the distribution of forces across the knee during physical activity [7,65]. An abnormal and excessive Q-angle causes an imbalance between the vastus medialis and vastus lateralis muscles, leading to an increased risk of knee problems, including patellofemoral pain syndrome, anterior knee pain, osteoarthritis, and degenerative knee disorders [66,67]. Athletes with an abnormal Q-angle can stabilize the knee joint by strengthening and stretching the muscles around the knee [68,69]. The quadriceps muscles, comprising the rectus femoris, vastus lateralis, vastus medialis, and vastus intermedius, are crucial for knee stability and function [70,71]. Thus, quadriceps muscle strength is required for a variety of athletic activities, including running, jumping, cutting movements, and landing movements [72,73]. Although exercises that can strengthen quadriceps muscles include squats, lunges, leg press, and leg extension [74], growing evidence shows that weight-bearing exercises such as knee extension weight-bearing exercise, hip flexion weight-bearing exercise, and hip abduction weight-bearing exercise are effective for athletes with an abnormal Q-angle [75].

Patellofemoral pain syndrome induced by a large Q-angle can result from core muscle instability because the dynamic imbalance of the torso and lower extremities can contribute to the development of patellofemoral pain [76]. Dynamic stability refers to the ability of the body to sustain its position or intended path despite external or internal disruptions [77,78]. The core of the body includes the spine, abdominal region, pelvis, hips, and proximal lower extremities [76,79]. Exercises that can strengthen the core muscles include cross curl-ups, side bridges, and quadrupedal stance [76].

## 14. Strengthening the Hip Muscles

Recently, strengthening the hip muscles has been suggested as an effective treatment for athletes with abnormal Q-angles [80,81]. Excessive hip movements in the frontal and transverse planes can place stress on the patellofemoral joint [82,83]. Weakness or imbalance in the hip muscles, including the gluteus medius, gluteus maximus, and hip abductors, can lead to excessive hip adduction and internal rotation, which contribute to an increased Q-angle and altered lower limb alignment [84,85]. Further, hip muscles are important for dynamic knee valgus, which is characterized by the inward collapse of the knee toward the middle of the body during functional movements [86,87]. Dynamic knee valgus is associated with an increased Q-angle and is a risk factor for various lower limb injuries, including patellofemoral pain syndrome [88]. Strengthening the hip abductors and external rotators can help control dynamic knee valgus by stabilizing the pelvis and controlling hip adduction and internal rotation, reducing the Q-angle [89]. Therefore, strong hip muscles play a significant role in stabilizing the pelvis and controlling hip movement [90,91]. Exercises that can strengthen the hip muscles include side-lying leg lifts and clamshells for hip adduction exercise, band walks and seated hip external rotation for hip external rotation exercise, bridge exercise and standing hip extension for hip extension exercise, standing hip flexion and supine hip flexion for hip flexion exercise, and standing hip adduction and side-lying hip adduction for hip adduction exercise [92,93,94].

## 15. Properly Warming up Prior to Exercise Can Decrease Risk

Warming up before exercise is essential for athletes with abnormal Q-angles to reduce the risk of muscle tension and injury [95]. A warm-up increases blood flow to the quadriceps muscles, raising muscle temperature [96]. This increased blood flow prepares the muscles, tendons, and ligaments for the demands of exercise, reducing the risk of strains and other injuries. Warming up stretches quadriceps muscles and tendons, improving range of motion [97]. The improved range of motion allows the muscles to lengthen and contract more efficiently during exercise, reducing the risk of muscle strains and tears, especially in athletes with abnormal Q-angles [98].

The female anatomy exhibits distinct biomechanical characteristics compared to the male anatomy, specifically concerning hip structure and movement patterns [99]. The anatomical difference includes a wider pelvis, a larger Q-angle, and looser ligaments [100]. For this reason, female athletes often face an increased risk of injury during sports activities [99]. It becomes imperative for female athletes to practice proper techniques to prevent the risk of injury. Landing mechanics are considered to prevent the risk of knee and hip injuries. A soft-landing technique with knees slightly bent and weight distributed across the entire foot, not just the heels, can reduce stress on the patella [101]. Jumping techniques that engage the glutes and core muscles can reduce stress on the quadriceps. Cutting and changing direction mechanics that improve footwork, hip rotation, and controlled deceleration can prevent imbalanced forces that stress the knee joint [102]. Exercises such as glute bridges, side-lying leg lifts, and clamshells that focus on hip strength and mobility improve pelvic alignment and reduce excessive inward collapse of the knees.

## 16. Challenges

One of the challenges for the Q-angle to gain clinical and therapeutic significance in the field is to establish accurate normal values for both genders in terms of different sports branches. In order to establish these values, other physical parameters such as age, femur length, height, and pelvic widths should be taken into consideration. Furthermore, one of the most frequently used clinical parameters for assessing the forces and other factors applied to the patellofemoral joint by the quadriceps, the Q-angle, is thought to be a good indicator of both sports performance, impact on the knee, and the presence of various patellofemoral disorders. It is critical to establish accurate values for the various patellofemoral disorders in order to facilitate the diagnosis and treatment of these conditions.

## 17. Limitations

One of the limitations of this review is the lack of recent articles on this topic, especially as it relates to the impact on athletic performance and risk of injuries in females. Also, a further limitation is the lack of inclusion of male data to enable a comparison in relation to athletic performance. In addition, there is a lack of studies on the impact of the Q-angle on different sports branches.

## 18. Conclusions

Women’s sports, including track, soccer, gymnastics, basketball, volleyball, and softball, are becoming more and more popular. However, the Q-angle is very important for determining how well female athletes perform and how likely they are to develop injuries. The factors that should be considered when screening female athletes for injuries have been highlighted in this review. Evaluating Q-angle changes early in younger populations and determining the basal Q-angle value may improve a clinician’s capacity to identify patients who are significantly at risk of developing patellofemoral disease. Studies show that athletes with higher Q-angles face multiple setbacks when compared to athletes with lower Q-angles. In addition to its impact on performance, a high Q-angle also increases the risk of injury by changing the biomechanics of the knee. Programs for sport education should place a strong emphasis on how the Q-angle affects female athletes’ performance. By knowing more about the Q-angle, clinicians, athletes, and coaches alike can be better prepared for competition and the setbacks and advantages of an individual’s Q-angle.

## Figures and Tables

**Table 1:** How the male and female average Q-angles—14° and 17°, respectively—influence the direction of force as well as the distribution of force. These forces were calculated using the male and female average Q-angles.

Displacement of Force Due to the Q-Angle			
Weight in kg	Type of Force	Male Force Output	Female Force Output
60	Exerted	61.86	62.76
	Extensor	60	60
	Lateral	14.52	17.52
80	Exerted	82.47	83.68
	Extensor	80	80
	Lateral	19.36	23.36
100	Exerted	103.09	104.60
	Extensor	100	100
	Lateral	24.20	29.20
120	Exerted	123.71	125.52
	Extensor	120	120
	Lateral	29.04	35.04
140	Exerted	144.32	146.44
	Extensor	140	140
	Lateral	33.88	40.88

## References

[1] Phatama K.Y., Isma S., Devi L.K., Siahaan L.D., Pribadi A., Pradana A.S., Mustasir E., Hidayat M. (2022). Relationship of Anterior Knee Pain with Quadriceps Angle and Anthropometric Measurements in an Asian Female Population. Malays. Orthop. J..

[2] Saç A., Taşmektepligil M.Y. (2018). Correlation between the Q angle and the isokinetic knee strength and muscle activity. Turk. J. Phys. Med. Rehabil..

[3] Brattstroem H. (1964). Shape of the intercondylar groove normally and in recurrent dislocation of patella. A clinical and X-ray-anatomical investigation. Acta Orthop. Scand. Suppl..

[4] Merchant A.C., Fraiser R., Dragoo J., Fredericson M. (2020). A reliable Q angle measurement using a standardized protocol. Knee.

[5] Johnson F., Leitl S., Waugh W. (1980). The distribution of load across the knee. A comparison of static and dynamic measurements. J. Bone Jt. Surg. Br..

[6] Shojaedin S.S., Letafatkar A., Hadadnezhad M., Dehkhoda M.R. (2014). Relationship between functional movement screening score and history of injury and identifying the predictive value of the FMS for injury. Int. J. Inj. Contr Saf. Promot..

[7] Loudon J.K. (2016). Biomechanics and pathomechanics of the patellofemoral joint. Int. J. Sports Phys. Ther..

[8] Skouras A.Z., Kanellopoulos A.K., Stasi S., Triantafyllou A., Koulouvaris P., Papagiannis G., Papathanasiou G. (2022). Clinical Significance of the Static and Dynamic Q-angle. Cureus.

[9] Milovanović D., Begović N., Bukva B., Dučić S., Vlahović A., Paunović Z., Kadija M., Topalović N., Stijak L. (2023). The Influence of the Q-Angle and Muscle Strength on Idiopathic Anterior Knee Pain in Adolescents. Medicina.

[10] Boling M., Padua D., Marshall S., Guskiewicz K., Pyne S., Beutler A. (2010). Gender differences in the incidence and prevalence of patellofemoral pain syndrome. Scand. J. Med. Sci. Sports.

[11] Sharma R., Vaibhav V., Meshram R., Singh B., Khorwal G. (2023). A Systematic Review on Quadriceps Angle in Relation to Knee Abnormalities. Cureus.

[12] Vora M., Curry E., Chipman A., Matzkin E., Li X. (2017). Patellofemoral pain syndrome in female athletes: A review of diagnoses, etiology and treatment options. Orthop. Rev..

[13] Sheehan F.T., Derasari A., Fine K.M., Brindle T.J., Alter K.E. (2010). Q-angle and J-sign: Indicative of maltracking subgroups in patellofemoral pain. Clin. Orthop. Relat. Res..

[14] Loudon J.K. (2016). The master female triathlete. Phys. Ther. Sport..

[15] Insall J., Falvo K.A., Wise D.W. (1976). Chondromalacia Patellae. A prospective study. J. Bone Jt. Surg. Am..

[16] Smith T.O., Hunt N.J., Donell S.T. (2008). The reliability and validity of the Q-angle: A systematic review. Knee Surg. Sports Traumatol. Arthrosc..

[17] Gwynne C.R., Curran S.A. (2018). Two-dimensional frontal plane projection angle can identify subgroups of patellofemoral pain patients who demonstrate dynamic knee valgus. Clin. Biomech..

[18] Silva D.e.O., Briani R.V., Pazzinatto M.F., Gonçalves A.V., Ferrari D., Aragão F.A., de Azevedo F.M. (2015). Q-angle static or dynamic measurements, which is the best choice for patellofemoral pain?. Clin. Biomech..

[19] Greene C.C., Edwards T.B., Wade M.R., Carson E.W. (2001). Reliability of the quadriceps angle measurement. Am. J. Knee Surg..

[20] Munro A., Herrington L., Carolan M. (2012). Reliability of 2-dimensional video assessment of frontal-plane dynamic knee valgus during common athletic screening tasks. J. Sport. Rehabil..

[21] Philp F., Leboeuf F., Pandyan A., Stewart C. (2019). “Dynamic knee valgus”—Are we measuring what we think we’re measuring? An evaluation of static and functional knee calibration methods for application in gait and clinical screening tests of the overhead squat and hurdle step. Gait Posture.

[22] Horwood A.M., Chockalingam N. (2017). Defining excessive, over, or hyper-pronation: A quandary. Foot.

[23] Powers C.M. (2010). The influence of abnormal hip mechanics on knee injury: A biomechanical perspective. J. Orthop. Sports Phys. Ther..

[24] Sutherland M.B., Langer B., Wassersug R. (2012). Getting a leg up on the competition: The importance of osteology in elite athletics. Am. J. Bioeth..

[25] Escamilla-Martínez E., Sánchez Martín F., Ramos-Ortega J., González-García P., Cortés-Vega M.D., Fernández-Seguín L.M. (2023). Age related changes in the Q angle of non-professional football players. Heliyon.

[26] Ahmetov I.I., Egorova E.S., Gabdrakhmanova L.J., Fedotovskaya O.N. (2016). Genes and Athletic Performance: An Update. Med. Sport. Sci..

[27] Grelsamer R.P., Klein J.R. (1998). The biomechanics of the patellofemoral joint. J. Orthop. Sports Phys. Ther..

[28] Lankhorst N.E., Bierma-Zeinstra S.M., van Middelkoop M. (2013). Factors associated with patellofemoral pain syndrome: A systematic review. Br. J. Sports Med..

[29] Percy E.C., Strother R.T. (1985). Patellalgia. Phys. Sportsmed..

[30] Huston L.J., Greenfield M.L., Wojtys E.M. (2000). Anterior cruciate ligament injuries in the female athlete. Potential risk factors. Clin. Orthop. Relat. Res..

[31] Mizuno Y., Kumagai M., Mattessich S.M., Elias J.J., Ramrattan N., Cosgarea A.J., Chao E.Y. (2001). Q-angle influences tibiofemoral and patellofemoral kinematics. J. Orthop. Res..

[32] van Gent R.N., Siem D., van Middelkoop M., van Os A.G., Bierma-Zeinstra S.M., Koes B.W. (2007). Incidence and determinants of lower extremity running injuries in long distance runners: A systematic review. Br. J. Sports Med..

[33] Tuna B.K., Semiz-Oysu A., Pekar B., Bukte Y., Hayirlioglu A. (2014). The association of patellofemoral joint morphology with chondromalacia patella: A quantitative MRI analysis. Clin. Imaging.

[34] Cavazzuti L., Merlo A., Orlandi F., Campanini I. (2010). Delayed onset of electromyographic activity of vastus medialis obliquus relative to vastus lateralis in subjects with patellofemoral pain syndrome. Gait Posture.

[35] Hvid I., Andersen L.I. (1982). The quadriceps angle and its relation to femoral torsion. Acta Orthop. Scand..

[36] Taunton J.E., Ryan M.B., Clement D.B., McKenzie D.C., Lloyd-Smith D.R., Zumbo B.D. (2002). A retrospective case-control analysis of 2002 running injuries. Br. J. Sports Med..

[37] Moul J.L. (1998). Differences in Selected Predictors of Anterior Cruciate Ligament Tears Between Male and Female NCAA Division I Collegiate Basketball Players. J. Athl. Train..

[38] Mancino F., Gabr A., Plastow R., Haddad F.S. (2023). Anterior cruciate ligament injuries in female athletes. Bone Jt. J..

[39] Ireland M.L. (1999). Anterior cruciate ligament injury in female athletes: Epidemiology. J. Athl. Train..

[40] Choudhary R., Malik M., Aslam A., Khurana D., Chauhan S. (2019). Effect of various parameters on Quadriceps angle in adult Indian population. J. Clin. Orthop. Trauma..

[41] Cerny K. (1995). Vastus medialis oblique/vastus lateralis muscle activity ratios for selected exercises in persons with and without patellofemoral pain syndrome. Phys. Ther..

[42] Cowan D.N., Jones B.H., Frykman P.N., Polly D.W., Harman E.A., Rosenstein R.M., Rosenstein M.T. (1996). Lower limb morphology and risk of overuse injury among male infantry trainees. Med. Sci. Sports Exerc..

[43] Shambaugh J.P., Klein A., Herbert J.H. (1991). Structural measures as predictors of injury basketball players. Med. Sci. Sports Exerc..

[44] Rauh M.J., Koepsell T.D., Rivara F.P., Rice S.G., Margherita A.J. (2007). Quadriceps angle and risk of injury among high school cross-country runners. J. Orthop. Sports Phys. Ther..

[45] Jafari A.A., Lotfi-Kamran M.H., Ghafoorzadeh M., Shaddel S.M. (2017). Evaluation of Surface Characteristics of Denture Base Using Organic-Inorganic Hybrid Coating: An SEM Study. J. Dent. Biomater..

[46] Araújo V.L., Souza T.R., Carvalhais V.O.D.C., Cruz A.C., Fonseca S.T. (2017). Effects of hip and trunk muscle strengthening on hip function and lower limb kinematics during step-down task. Clin. Biomech..

[47] Lankhorst N.E., Bierma-Zeinstra S.M., van Middelkoop M. (2012). Risk factors for patellofemoral pain syndrome: A systematic review. J. Orthop. Sports Phys. Ther..

[48] Nascimento L.R., Teixeira-Salmela L.F., Souza R.B., Resende R.A. (2018). Hip and Knee Strengthening Is More Effective Than Knee Strengthening Alone for Reducing Pain and Improving Activity in Individuals with Patellofemoral Pain: A Systematic Review With Meta-analysis. J. Orthop. Sports Phys. Ther..

[49] Schilke J.M., Johnson G.O., Housh T.J., O’Dell J.R. (1996). Effects of muscle-strength training on the functional status of patients with osteoarthritis of the knee joint. Nurs. Res..

[50] Sahabuddin F.N.A., Jamaludin N.I., Amir N.H., Shaharudin S. (2021). The effects of hip- and ankle-focused exercise intervention on dynamic knee valgus: A systematic review. PeerJ.

[51] Coudeyre E., Jegu A.G., Giustanini M., Marrel J.P., Edouard P., Pereira B. (2016). Isokinetic muscle strengthening for knee osteoarthritis: A systematic review of randomized controlled trials with meta-analysis. Ann. Phys. Rehabil. Med..

[52] Miao P., Xu Y., Pan C., Liu H., Wang C. (2015). Vastus medialis oblique and vastus lateralis activity during a double-leg semisquat with or without hip adduction in patients with patellofemoral pain syndrome. BMC Musculoskelet. Disord..

[53] El Sawy M.M., Mikkawy D.M.E., El-Sayed S.M., Desouky A.M. (2021). Morphometric analysis of vastus medialis oblique muscle and its influence on anterior knee pain. Anat. Cell Biol..

[54] Sakai N., Luo Z.-P., Rand J.A., An K.-N. (2000). The influence of weakness in the vastus medialis oblique muscle on the patellofemoral joint: An in vitro biomechanical study. Clin. Biomech..

[55] Pal S., Draper C.E., Fredericson M., Gold G.E., Delp S.L., Beaupre G.S., Besier T.F. (2011). Patellar maltracking correlates with vastus medialis activation delay in patellofemoral pain patients. Am. J. Sports Med..

[56] Elias J.J., Kilambi S., Goerke D.R., Cosgarea A.J. (2009). Improving vastus medialis obliquus function reduces pressure applied to lateral patellofemoral cartilage. J. Orthop. Res..

[57] Khoshkhoo M., Killingback A., Robertson C.J., Adds P.J. (2016). The effect of exercise on vastus medialis oblique muscle architecture: An ultrasound investigation. Clin. Anat..

[58] Edson C.J. (2015). Rehabilitation following PCL reconstruction: Scientific and theoretical basis. Posterior Cruciate Ligament Inj. A Pract. Guide Manag..

[59] Zhang X., Hu M., Lou Z., Liao B. (2017). Effects of strength and neuromuscular training on functional performance in athletes after partial medial meniscectomy. J. Exerc. Rehabil..

[60] Coqueiro K.R., Bevilaqua-Grossi D., Bérzin F., Soares A.B., Candolo C., Monteiro-Pedro V. (2005). Analysis on the activation of the VMO and VLL muscles during semisquat exercises with and without hip adduction in individuals with patellofemoral pain syndrome. J. Electromyogr. Kinesiol..

[61] Chang W.D., Huang W.S., Lai P.T. (2015). Muscle Activation of Vastus Medialis Oblique and Vastus Lateralis in Sling-Based Exercises in Patients with Patellofemoral Pain Syndrome: A Cross-Over Study. Evid. Based Complement. Alternat Med..

[62] Stensdotter A.K., Hodges P.W., Mellor R., Sundelin G., Häger-Ross C. (2003). Quadriceps activation in closed and in open kinetic chain exercise. Med. Sci. Sports Exerc..

[63] Khasawneh R.R., Allouh M.Z., Abu-El-Rub E. (2019). Measurement of the quadriceps (Q) angle with respect to various body parameters in young Arab population. PLoS ONE.

[64] Chhabra P.K., Godwin R., Setiya M. (2016). “Quadriceps angle”: An important indicator of biomechanical function of lower extremity and its relation with anterior knee pain. Int. J. Sci. Study.

[65] Galea A., Albers J. (1994). Patellofemoral pain: Targeting the cause. Phys. Sports Med..

[66] Sendur O.F., Gurer G., Yildirim T., Ozturk E., Aydeniz A. (2006). Relationship of Q angle and joint hypermobility and Q angle values in different positions. Clin. Rheumatol..

[67] Honarpishe R., Bakhtiary A.H., Olyaei G. (2015). Effect of quadriceps exercise training on muscle fiber angle in patients with patellofemoral pain syndrome. Middle East J. Rehabil. Health.

[68] Fendri T., Beaune B., Kasmi S., Chaari F., Sahli S., Boyas S. (2024). Relationship Between Postural Stability and Proprioception, Pain, Quadriceps Strength, and Muscle Tightness in Athletes With Patellar Tendinopathy. Sports Health.

[69] Abulhasan J.F., Grey M.J. (2017). Anatomy and physiology of knee stability. J. Funct. Morphol. Kinesiol..

[70] Keays S.L., Bullock-Saxton J., Newcombe P., Keays A. (2003). The relationship between knee strength and functional stability before and after anterior cruciate ligament reconstruction. J. Orthop. Res..

[71] Willigenburg N.W., McNally M.P., Hewett T.E. (2014). Quadriceps and hamstrings strength in athletes. Hamstring and Quadriceps Injuries in Athletes: A Clinical Guide.

[72] Schmitt L.C., Paterno M.V., Hewett T.E. (2012). The impact of quadriceps femoris strength asymmetry on functional performance at return to sport following anterior cruciate ligament reconstruction. J. Orthop. Sports Phys. Ther..

[73] Dutton R.A., Khadavi M.J., Fredericson M. (2014). Update on rehabilitation of patellofemoral pain. Curr. Sports Med. Rep..

[74] Lee J., Lee H., Lee W. (2014). Effect of Weight-bearing Therapeutic Exercise on the Q-angle and Muscle Activity Onset Times of Elite Athletes with Patellofemoral Pain Syndrome: A Randomized Controlled Trial. J. Phys. Ther. Sci..

[75] Chevidikunnan M.F., Al Saif A., Gaowgzeh R.A., Mamdouh K.A. (2016). Effectiveness of core muscle strengthening for improving pain and dynamic balance among female patients with patellofemoral pain syndrome. J. Phys. Ther. Sci..

[76] Kwon Y.J., Park S.J., Jefferson J., Kim K. (2013). The effect of open and closed kinetic chain exercises on dynamic balance ability of normal healthy adults. J. Phys. Ther. Sci..

[77] Zazulak B.T., Hewett T.E., Reeves N.P., Goldberg B., Cholewicki J. (2007). Deficits in neuromuscular control of the trunk predict knee injury risk: Prospective biomechanical-epidemiologic study. Am. J. Sports Med..

[78] Chung E.-J., Kim J.-H., Lee B.-H. (2013). The effects of core stabilization exercise on dynamic balance and gait function in stroke patients. J. Phys. Ther. Sci..

[79] Santos T.R., Oliveira B.A., Ocarino J.M., Holt K.G., Fonseca S.T. (2015). Effectiveness of hip muscle strengthening in patellofemoral pain syndrome patients: A systematic review. Braz. J. Phys. Ther..

[80] Ireland M.L., Willson J.D., Ballantyne B.T., Davis I.M. (2003). Hip strength in females with and without patellofemoral pain. J. Orthop. Sports Phys. Ther..

[81] Barton C.J., Lack S., Malliaras P., Morrissey D. (2013). Gluteal muscle activity and patellofemoral pain syndrome: A systematic review. Br. J. Sports Med..

[82] Mascal C.L., Landel R., Powers C. (2003). Management of patellofemoral pain targeting hip, pelvis, and trunk muscle function: 2 case reports. J. Orthop. Sports Phys. Ther..

[83] Hollman J.H., Kolbeck K.E., Hitchcock J.L., Koverman J.W., Krause D.A. (2006). Correlations between hip strength and static foot and knee posture. J. Sport. Rehabil..

[84] Alzahrani A.M., Alzhrani M., Alshahrani S.N., Alghamdi W., Alqahtani M., Alzahrani H. (2021). Is hip muscle strength associated with dynamic knee valgus in a healthy adult population? A systematic review. Int. J. Environ. Res. Public Health.

[85] Dix J., Marsh S., Dingenen B., Malliaras P. (2019). The relationship between hip muscle strength and dynamic knee valgus in asymptomatic females: A systematic review. Phys. Ther. Sport.

[86] Almeida G.P.L., França F.J.R., Magalhães M.O., Burke T.N., Marques A.P. (2016). Q-angle in patellofemoral pain: Relationship with dynamic knee valgus, hip abductor torque, pain and function. Rev. Bras. De Ortop..

[87] Wilczyński B., Wąż P., Zorena K. (2021). Impact of three strengthening exercises on dynamic knee valgus and balance with poor knee control among young football players: A randomized controlled trial. Healthcare.

[88] Ismail M.M., Gamaleldein M., Hassa K. (2013). Closed kinetic chain exercises with or without additional hip strengthening exercises in management of patellofemoral pain syndrome: A randomized controlled trial. Eur. J. Phys. Rehabil. Med..

[89] Nakagawa T.H., Muniz T.B., Baldon R.d.M., Dias Maciel C., de Menezes Reiff R.B., Serrão F.V. (2008). The effect of additional strengthening of hip abductor and lateral rotator muscles in patellofemoral pain syndrome: A randomized controlled pilot study. Clin. Rehabil..

[90] Lee J.-H., Cynn H.-S., Choi S.-A., Yoon T.-L., Jeong H.-J. (2013). Effects of different hip rotations on gluteus medius and tensor fasciae latae muscle activity during isometric side-lying hip abduction. J. Sport Rehabil..

[91] Youdas J.W., Foley B.M., Kruger B.L., Mangus J.M., Tortorelli A.M., Madson T.J., Hollman J.H. (2013). Electromyographic analysis of trunk and hip muscles during resisted lateral band walking. Physiother. Theory Pract..

[92] Contreras B.M., Cronin J.B., Schoenfeld B.J., Nates R.J., Sonmez G.T. (2013). Are All Hip extension exercises created equal?. Strength Cond. J..

[93] Pohl P. (2013). The Patriot Program: A Dynamic Warm-Up for the Risk Reduction of Anterior Cruciate Ligament Injureis in High School Female Basketball Players.

[94] Aguilar A.J., DiStefano L.J., Brown C.N., Herman D.C., Guskiewicz K.M., Padua D.A. (2012). A dynamic warm-up model increases quadriceps strength and hamstring flexibility. J. Strength. Cond. Res..

[95] Kovacs M. (2009). Dynamic Stretching: The Revolutionary New Warm-Up Method to Improve Power, Performance and Range of Motion.

[96] Rabin A., Kozol Z. (2010). Measures of range of motion and strength among healthy women with differing quality of lower extremity movement during the lateral step-down test. J. Orthop. Sports Phys. Ther..

[97] Wang S.C., Brede C., Lange D., Poster C.S., Lange A.W., Kohoyda-Inglis C., Sochor M.R., Ipaktchi K., Rowe S.A., Patel S. (2004). Gender differences in hip anatomy: Possible implications for injury tolerance in frontal collisions. Annual Proceedings/Association for the Advancement of Automotive Medicine.

[98] Smith F.W., Smith P.A. (2002). Musculoskeletal differences between males and females. Sports Med. Arthrosc. Rev..

[99] Tamura A., Akasaka K., Otsudo T. (2021). Contribution of lower extremity joints on energy absorption during soft landing. Int. J. Environ. Res. Public Health.

[100] Sheykhi S., Norasteh A.A. (2019). Comparison of different methods of core muscle fatigue on lower extremity function tests among female athletes. Tabari Biomed. Stud. Res. J..

[101] Kim C.-M., Kong Y.-S., Hwang Y.-T., Park J.-W. (2018). The effect of the trunk and gluteus maximus muscle activities according to support surface and hip joint rotation during bridge exercise. J. Phys. Ther. Sci..

[102] Kang M.-H., Kim S.-Y., Yu I.-Y., Oh J.-S. (2019). Effects of real-time visual biofeedback of pelvic movement on electromyographic activity of hip muscles and lateral pelvic tilt during unilateral weight-bearing and side-lying hip abduction exercises. J. Electromyogr. Kinesiol..

